# Eco-Design and Characterization of Sustainable Lightweight Gypsum Composites for Panel Manufacturing including End-of-Life Tyre Wastes

**DOI:** 10.3390/ma17030635

**Published:** 2024-01-28

**Authors:** Daniel Ferrández, Manuel Álvarez, Alicia Zaragoza-Benzal, Paulo Santos

**Affiliations:** 1 Departamento de Tecnología de la Edificación, Escuela Técnica Superior de Edificación, Avda. Juan de Herrera, nº 6, 28040 Madrid, Spain; daniel.fvega@upm.es (D.F.); manuel.alvarezd@upm.es (M.Á.); alicia.zaragoza@upm.es (A.Z.-B.); 2 ISISE, ARISE, Department of Civil Engineering, University of Coimbra, 3030-788 Coimbra, Portugal

**Keywords:** gypsum composites, end-of-life tyres (ELT), recycled, characterisation, precast, circular economy

## Abstract

The incorporation of rubber recycled aggregates from end-of-life tyres (ELT) in the manufacturing process of sustainable building materials has gained great interest in recent decades as a result of the large volume of this waste being generated annually. In this work, the objective is to make a contribution towards the circularity of construction products by carrying out a physico-mechanical characterisation of new gypsum composites made with the incorporation of these recycled rubber aggregates. To this end, up to 30% by volume of the original raw material has been substituted, analysing the mechanical resistance to bending and compression. Although lower than those of traditional gypsum material, both properties exceed the limits set at 1 and 2 MPa, respectively, by the current regulations. In addition, water absorption by capillarity significantly decreases, and thermal conductivity is reduced by more than 35% with respect to the reference material. Finally, in order to provide the research with a practical application, a prefabricated plate design has been proposed that incorporates the gypsum materials studied and an agglomerated rubber band that increases the thermal resistance and improves the efficiency of the designed construction system. In this way, this research reflects the potential of these novel building materials and explores new avenues for their application in building construction.

## 1. Introduction

In general terms, it is estimated that at least one tyre per inhabitant is discarded in the most economically developed countries [1]. The volume of tyre waste spread across the globe reaches figures around 1.5 billion end-of-life tyres (ELTs) per year [2]. In the European Union alone, around 4.5 million tonnes are produced annually, ranking third in the world, behind only the United States (5.2 million tonnes) and China (14.6 million tonnes) [3]. These alarming figures have led governments in different countries to include the management of this waste as one of the major challenges worldwide, with the aim of mitigating its environmental impact and reducing its high generation volumes [4].

Regarding the current management of this ELT waste, Abbas-Abadi et al. reported that only 3–15% of these wastes are recycled, 5–23% are reused, 20–30% are landfilled and 25–60% are mostly incinerated [5]. The incineration technique for energy recovery is widely used in industry and is a highly environmentally damaging strategy, as 450 kg of toxic gases (such as CO, SO_2_, NO_2_ HCl, butadiene and aromatic substances [6]) and 270 kg of soot are produced for every tonne of ELT incinerated, leading to the accumulation of suspended particles in the air and the contamination of aquifers [7,8]. The accumulation in landfills also poses a risk due not only to their visual impact but also to the high flammability of these solid wastes, which can ignite fires that are difficult to extinguish [9].

For all these reasons, and especially during the last 20 years, there has been an increase in studies attempting to reincorporate these solid wastes from ELT into new sustainable building materials [10]. The incorporation of ELT rubber wastes in gypsum composites has been studied with the purpose of improving some of their physical properties, such as their thermoacoustic behaviour or reducing the capillary water absorption capacity [11,12]. Although, in general, gypsum is a widely used material in the construction sector, it has been used since ancient times for the production of cladding and interior finishes [13]. Although it has excellent performance in the hygrothermal regulation of living spaces [14], as well as a clean and satin appearance after in situ application [15], the industrialisation of the construction sector has been the key driver for its large-scale development through new prefabricated systems [16].

In this regard, many studies have been carried out in recent years trying to incorporate plastic waste into gypsum composites under circular economy criteria. Thus, additions of polypropylene from coffee capsules [17], thermal insulation waste [18] or low-density polyethylene from recycled bags [19] have been explored. All of them have demonstrated their suitability to produce sustainable and technically feasible prefabricated construction products. Table 1 shows a synthesis of the studies carried out in the last three years on gypsum plasters with rubber additions and the properties analysed in them. For this purpose, a search has been carried out in Web of Science, including the keywords ((“Gypsum*” OR “Plaster*”) AND “Rubber”).

As a result of the studies included in Table 1, it can be seen that a large part of the research carried out analyses the physical–mechanical behaviour of gypsum composites with rubber additions. In this sense, the aim is to find an optimum ratio between the amount of recycled material added to reduce the final density of the materials and to obtain mechanical strengths in accordance with the standards [28]. This sometimes leads to the addition of reinforcement fibres to improve ductility [20] or to the design of prefabricated “sandwich”-type panels with sheets that provide greater mechanical stability to the designed plate or panel [22,23]. On the other hand, a large number of studies analysed the improvements in the thermal resistance and acoustic behaviour of the composites produced [24], which encourages their application in construction to improve energy efficiency and the habitability conditions of buildings [25]. Finally, it should be noted that these rubber recycled materials, although they have a beneficial effect in reducing capillary water absorption [12], sometimes reduce the fire resistance characteristic of gypsum composites [21].

The main objective of this work is to carry out a physico-mechanical characterisation of the gypsum composites produced by partially substituting the original raw material with the fine fraction of recycled rubber from ELT ranging from 0–0.8 mm. To this end, an experimental campaign has been designed that includes mechanical resistance tests in accordance with the current reference standards, behaviour against the action of water and a study of its thermal behaviour, among others. Once the initial campaign has been completed, a novel prefabricated product is proposed for the construction of interior partitions and wall cladding made of the different plaster composites analysed and a strip of agglomerated recycled rubber. Moulds have been designed to produce these sustainable prefabricated products to scale, with the aim of studying their most relevant properties for building and assessing their suitability for the construction of interior partitions. This is, therefore, an original work in line with the objectives to promote the circularity of construction products included in the European Green Pact [29].

## 2. Materials and Methods

In this section, the raw materials used for the elaboration of the different gypsum compounds are presented, together with the sample preparation process and a description of the experimental programme carried out.

### 2.1. Materials

For the development of this research, fine gypsum was used as a binder material mixed with drinking water. In addition, two recycled rubber products from ELT were used: fine aggregates and pressed sheets.

#### 2.1.1. Binder and Water

For this study, type B1 building gypsum was used according to the classification in the UNE-EN 13279-1: 2009 standard [30]. This is a conglomerating material commonly used in building construction for interior cladding and interior partitions. Its most relevant characteristics, provided by the Saint-Gobain Placo Ibérica, S.A. group (Madrid, Spain), are listed in Table 2.

For the kneading process, drinking water from Canal de Isabel II (Madrid, Spain) in compliance with Council Directive 98/83/EC [33] was used. This type of water has been successfully used in previous research [25] and is characterised by its medium hardness and neutral pH.

#### 2.1.2. End-of-Life Tyres Products

**Figure 1:**
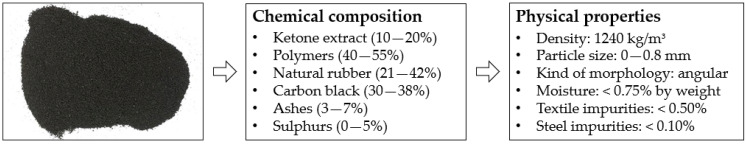
Physicochemical properties of the rubber aggregates from the ELTs used [12].

Firstly, rubber aggregates were used as a secondary raw material for the partial volume replacement of traditional gypsum. This powdery material has a particle diameter between 0 and 0.8 mm. Figure 1 shows their appearance, chemical composition and the most relevant physical properties.

**Figure 2:**
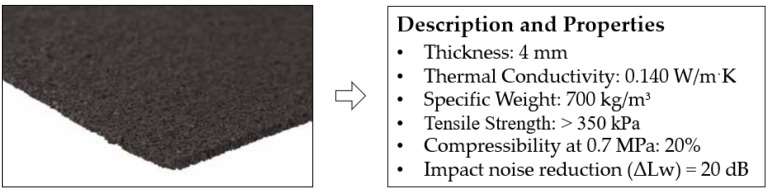
Properties of agglomerated recycled rubber provided by the manufacturer [34].

On the other hand, agglomerated recycled rubber strips have been used for the production of prefabricated ceiling panels with improved thermal performance. The main characteristics of these recycled materials are shown in Figure 2 and were provided by the company Corticeira Amorim, S.A. (Mozelos, Portugal).

### 2.2. Sample Preparation Process

For the preparation of the different gypsum composites studied in this research, the method and times recommended in the UNE-EN 13279-2:2014 standard were followed [35]. It should be noted that the composites were prepared by making progressive partial substitutions of the traditional plaster material by rubber recycled aggregates until reaching a 30% volume replacement of the original raw material. The water content of the samples was experimentally set to obtain a workable consistency, which was achieved with a paste diameter (water/plaster) of 165 ± 5 mm in the shaking table test.

Table 3 shows the proportions, both in mass and volume, used to produce the gypsum composites developed.

The curing process for the gypsum materials studied was as follows: First, the samples were kept in laboratory conditions (20 ± 2 °C temperature and 50 ± 5% relative humidity) for six days; then, during the 24 h prior to the tests, the samples were placed in an oven under the conditions set out in the UNE-EN 13279-2 standard of 40 ± 2 °C temperature and 50 ± 5% relative humidity. In this way, all the compounds were tested after seven days under the same starting conditions.

**Figure 3:**
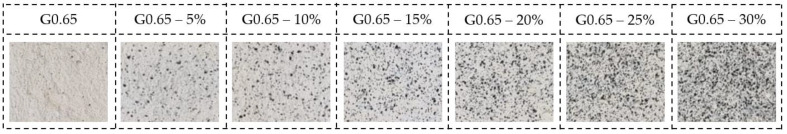
Cross-sections showing the different matrixes of the composites produced in this research.

Figure 3 shows the appearance of the prepared gypsum composite matrixes when cross-sectioned.

### 2.3. Experimental Programme

**Figure 4:**
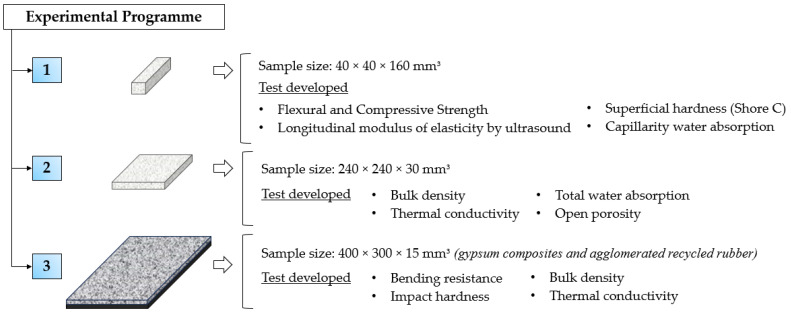
Diagram of the experimental programme developed in this research.

The experimental programme developed in this research is divided into three stages: (1) mechanical characterisation of the gypsum composites produced, (2) study of their most relevant physical properties and, finally, (3) design and characterisation of new sustainable prefabricated products. Figure 4 shows a scheme of the experimental process designed in relation to the size of the samples tested.

**Figure 5:**
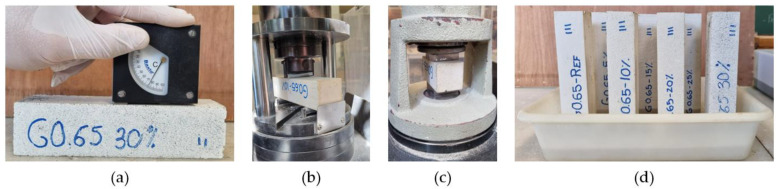
Images taken from tests carried out in the experimental programme: (**a**) surface hardness; (**b**) flexural strength; (**c**) compressive strength; (**d**) capillarity test.

Regarding the first phase of mechanical characterisation, a total of three 4 × 4 × 16 cm^3^ samples were tested for each type of dosage developed. Firstly, the longitudinal modulus of elasticity was determined using a Matest model C368 ultrasound machine (S.A.E. Ibertest, Madrid, Spain), equipped with emission–reception transducers working at 55 kHz. Vaseline was used at the junction interface to ensure adequate contact between the transducers and the samples. The surface hardness was determined with the aid of a Shore C hardness tester (Figure 5a) (S.A.E. Ibertest, Madrid, Spain), taking five measurements for each longitudinal face of the samples that had been in contact with the mould, according to the recommendations of the UNE 102042:2023 standard [36]. Finally, the flexural and compressive mechanical strengths were determined with the aid of an Ibertest hydraulic press (AUTOTEST 200-10SW) (S.A.E. Ibertest, Madrid, Spain), working with load speeds of 10 N/s and 20 N/s, respectively, until the specimens broke according to the recommendations of the UNE-EN 13279-2 standard [35] (Figure 5b,c).

In order to better understand the microstructure of the gypsum composites, scanning electron microscopy images were taken to observe the interaction between the rubber aggregates and the gypsum matrix. A Jeol JSM-820 microscope (Jeol, Croissy-sur-Seine, France) operating at 20 kV and equipped with Oxford EDX analysis was used for this test.

On the other hand, physical characterisation tests were carried out on the composites, first determining the bulk density as the ratio between the mass and volume of the 4 × 4 × 16 cm^3^ samples, in accordance with the procedure indicated in the UNE 102042:2023 standard [36]. At the same sample size, the capillary water absorption of the gypsum materials produced following the adaptation of UNE-EN 1925:1999 [37] was studied (Figure 5d). On the other hand, using samples of size 240 × 240 × 30 mm^3^, the thermal conductivity coefficient of the composites was determined with the help of a mini Hot-Box (DEC-FCTUC, Coimbra, Portugal) equipped with thermocouples. Finally, the total water absorption coefficient according to UNE-EN 14617-1:2013 [38] and the open porosity according to UNE-EN 1936:2007 [39] were determined for the same previously dried samples.

Finally, flexural strength and impact hardness tests were carried out on 400 × 300 × 15 mm^3^ plates composed of the developed gypsum materials and a sheet of agglomerated recycled rubber. The flexural strength test was carried out following the indications of the UNE-EN 12859:2012 standard [40] on a total of three samples, and the impact resistance was determined according to the procedure included in the same standard, measuring the diameter produced by a steel ball of 50 mm in diameter and a drop height of 50 cm.

## 3. Results

In this section, the results obtained for the different tests conducted in the experimental programme designed are presented, including a critical discussion of them and exploring their application possibilities.

### 3.1. Mechanical Characterisation Tests

**Figure 6:**
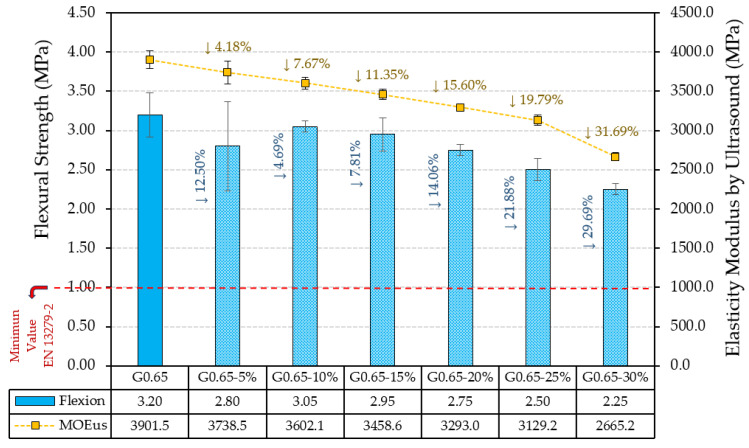
Flexural strength and longitudinal modulus of elasticity by ultrasounds.

Firstly, Figure 6 shows the values obtained for the bending strength and longitudinal modulus of elasticity by ultrasound for the different composites.

It can be observed how the progressive substitution of plaster material by recycled rubber aggregates causes a progressive decrease in the flexural strength of the gypsum composites. This same effect is observed in the longitudinal modulus of elasticity determined by ultrasound and presented in Figure 6. However, although, for the composite with the highest content of recycled material (G0.65-30%), a decrease in flexural strength of more than 29% is produced in comparison with the reference composite (G0.65), in all the cases analysed, the minimum values required by the standards for this mechanical property, set at 1 MPa, were exceeded. Thus, all the composites included in this study are suitable for use in buildings in accordance with the UNE-EN 13279-2:2014 standard.

**Figure 7:**
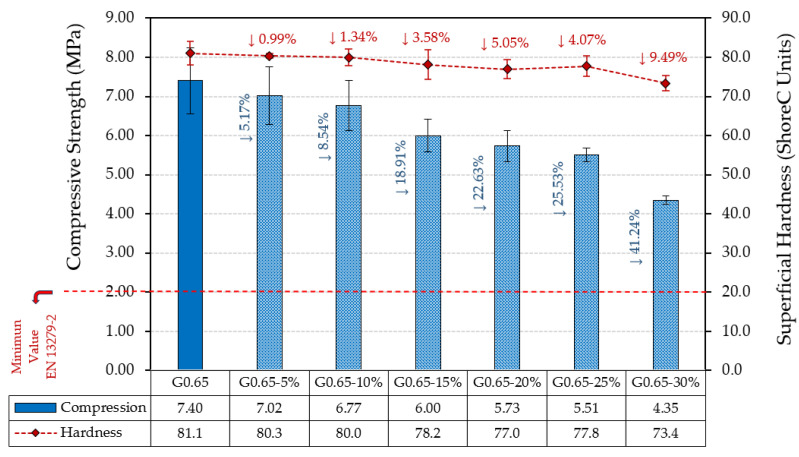
Compressive strength and surface hardness in Shore C.

On the other hand, Figure 7 shows the results obtained for the compressive strength and surface hardness of the developed gypsum materials.

As in Figure 6, the compressive strength results shown in Figure 7 reflect a progressive decrease in compressive strength as the amount of residue added increases. Thus, the G0.65-30% composite showed a decrease in compressive strength of up to 41% compared to the traditional G0.65 gypsum material. However, as in the case of flexural strength, all the composites analysed exceeded the minimum value required by the UNE-EN 13290-2 standard for use in buildings, which is set at 2 MPa. In the same way, surface hardness also experienced a slight decrease with the incorporation of ELT rubber waste in the production of the gypsum composites. The most unfavourable decrease was 9.49% for sample G0.65-30% with respect to the reference composite G0.65.

The results obtained for the mechanical properties analysed agree with those obtained previously by other researchers. Thus, Herrero et al. observed that, for partial substitutions in volume of the original gypsum material by rubber recycled aggregates of more than 50%, it is not possible to guarantee compliance with the minimum flexural and compressive strengths established in the standards [11]. A similar effect on the decrease in mechanical strength was observed by Vidales et al., who added plastic waste from electrical cables [41], and by Asadi et al., who incorporated waste from the automotive sector to produce gypsum mortars under circular economy criteria [42]. The relationship between the decrease in flexural strength and the modulus of elasticity, as well as between the compressive strength and surface hardness, has been observed in previous studies using textile fibres from ELT [43]. Finally, it should be noted that the incorporation of rubber wastes in other investigations has led to a significant decrease in the surface hardness of gypsum composites. In this regard, Jimenez-Rivero et al. observed a reduction in surface hardness compared to the reference composites of more than 20% when rubber waste was added at 7.5% by weight of the original gypsum material [44].

**Figure 8:**
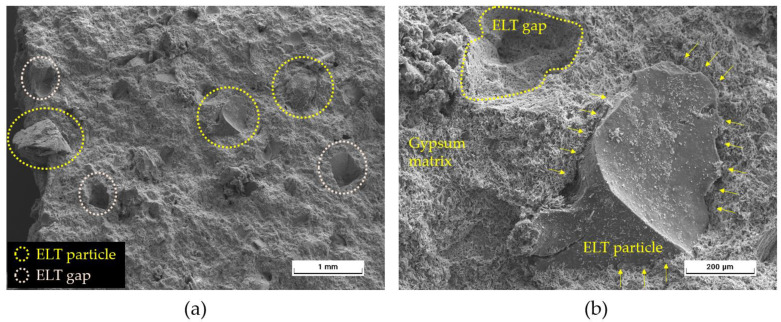
SEM images obtained for sample G0.65-20%. (**a**) Overview of the matrix of the processed composites. (**b**) Details of the adhesion between the ELT rubber aggregates and the plaster composite matrix.

Finally, in order to better understand the internal microstructure of the gypsum composites developed in this research, images were obtained by scanning electron microscopy (SEM). Figure 8 shows the interaction between the ELT aggregates and the gypsum matrix at different magnifications.

Figure 8a corresponds to a generic view of the plaster composite matrix after the flexural fracture test. Although a good integration of the residue in the matrix can be seen, voids produced by the detachment of the rubber after mechanical testing can be visualised. In Figure 8b, the characteristic acicular morphology of the dihydrate crystals (CaSO_4_∙2H_2_O) formed during the setting process of the gypsum material [45] envelop the rubber recycled aggregates, forming a compact matrix without chemical reactions at the interface. This corroborates what Herrero del Cura observed in his doctoral thesis, the adequate cohesion between the traditional gypsum material and the rubber particles from ELT [46].

### 3.2. Physical Characterisation Tests

**Figure 9:**
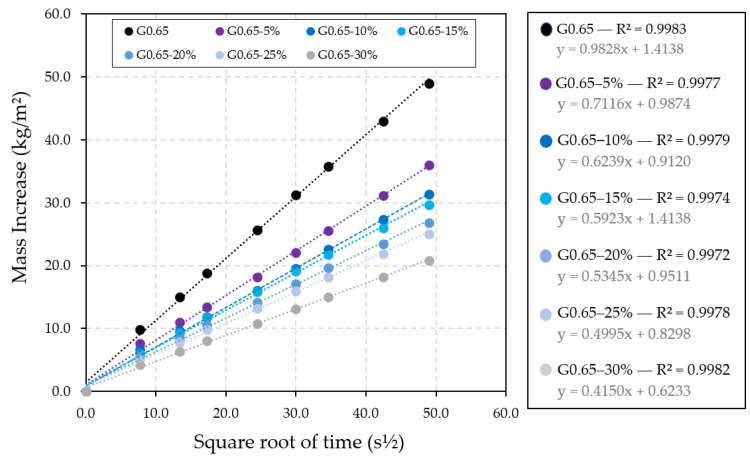
Evolution of capillary water absorption as a function of time.

For this physical characterisation, firstly, the results obtained for the behaviour of the gypsum composites analysed in relation to the action of water are presented. The effect of water absorption by capillarity was studied in the different materials developed, obtaining the results shown in Figure 9.

Figure 9 shows the evolution of the mass water absorption per unit area versus test time. Thus, it can be seen how the slope of the lines generated decreases as the recycled raw material content increases, which implies a decrease in capillary water absorption as a consequence of the incorporation of rubber recycled aggregates in the gypsum composite matrix. Thus, the compounds with the highest ELT waste content, sample G0.65-30%, showed the lowest water absorption during the 40-min test duration. Figure 9 shows the good correlation between water absorption and the course of time in s^½^, being, in all the cases, a linear adjustment with an R^2^ coefficient close to unity. This excellent correlation and beneficial effect of rubber recycled aggregates was observed, in part, in the research carried out by Zaldívar et al. [12].

Table 4 presents the results obtained for the open porosity of the materials analysed and the total water absorption test.

As can be seen in Table 4, both the open porosity and the total water absorption coefficient are reduced by incorporating rubber residues from ELT into the gypsum matrix. This effect is related to the decrease in capillary water absorption presented in Figure 9. In previous research [47,48], a similar effect on these properties has been observed when incorporating shredded plastic waste into gypsum composites. This effect is due to the lack of water absorption capacity of these rubber materials, which makes it difficult for water to rise through the gypsum composite by capillarity.

**Figure 10:**
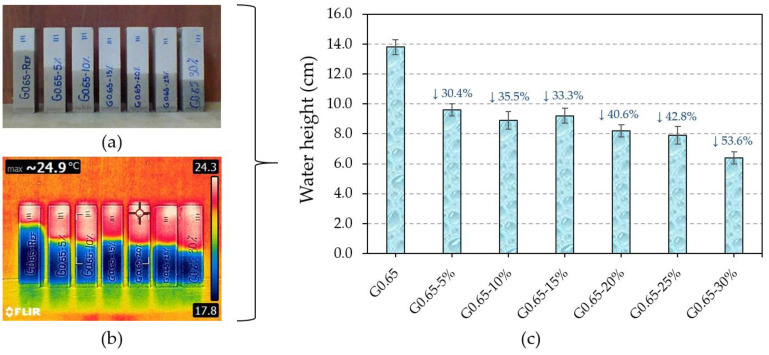
Capillarity test: (**a**) samples after the capillary water absorption test; (**b**) thermography of the samples tested; (**c**) maximum height reached by water in the 4 × 4 × 16 cm^3^ samples after the capillary test.

In the same way, the maximum height reached by the water after the capillarity test was analysed. These results are shown in Figure 10, which shows a progressive decrease in the final height reached by the water in the gypsum composites as the recycled raw material content increases. This effect is due to the larger pore diameter of the gypsum composites as the recycled rubber content increases, in accordance with Jurin’s Law.

As shown in Figure 10c and reflected in Figure 10a and thermography (Figure 10b), there is a reduction in the water height reached of up to 53.6% for the G0.65-30% composite versus the traditional gypsum material.

**Figure 11:**
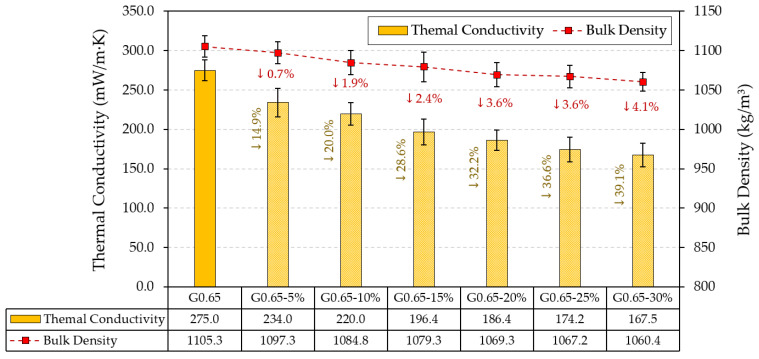
Thermal conductivity and bulk density results of the processed composites.

On the other hand, the effect of recycled rubber aggregates on the thermal conductivity and bulk density of gypsum composites is studied in Figure 11.

Figure 11 shows how the incorporation of ELT aggregates leads to a decrease in the thermal conductivity of the gypsum composites and their bulk density. Although it is true that, for the composite with the highest recycled raw material content (G0.65-30%), a slight lightening of the gypsum material of 4.1% occurs, its effect on the reduction in thermal conductivity is denoted by up to 39.1% with respect to the G0.65 reference material. This suggests the suitability of these composites for the production of prefabricated products for a sustainable and more energy-efficient building. This effect is due to the lower coefficient of thermal conductivity of rubber aggregates compared to traditional gypsum composites, as it has been observed in other works that the difference in thermal conductivity between the two types of raw materials can be up to 44% [11].

The results obtained in this research are in agreement with those observed by other researchers who have added plastic wastes to the matrix of gypsum composites. Examples include Pedreño-Rojas et al., who added polycarbonate waste and obtained a 16.9% decrease in thermal conductivity with respect to the reference [49], Vidales et al., with a 7.4% reduction in plaster composites with the addition of electrical cable insulation waste [50], or Ferrández et al., with a decrease of up to 18% in the thermal conductivity of gypsum composites with partial replacement of the original material with low-density polyethylene granules [19]. Regarding studies with rubber recycled aggregates from ELT, it has been observed that the incorporation of these wastes reduces the thermal conductivity of hardened gypsum composites. Thus, Herrero et al., in their work, obtained decreases in thermal conductivity of more than 25% in composites with partial replacement of the gypsum material by ELT aggregates of a size between 0 and 0.8 mm [46]. Similarly, the incorporation of other secondary raw materials from ELT, such as textile fibres in the manufacture of gypsum composites, has also led to a decrease in the final thermal conductivity of the materials developed and an improvement in their energy efficiency [43].

### 3.3. Design and Characterisation of New Sustainable Prefabricated Products

**Figure 12:**
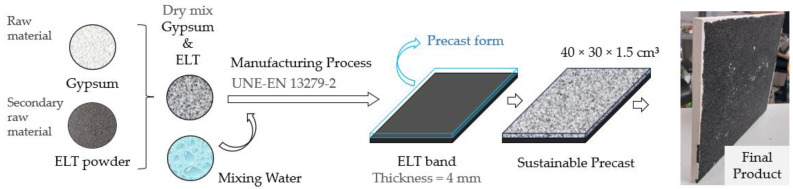
Production process diagram of the gypsum prefabricated products developed in this research.

The aim of this section is to show the application possibilities of the gypsum-based composite materials developed in this research. To this end, the design of a prefabricated panel for modular construction with a mixed composition of agglomerated recycled rubber strip and the different gypsum-based materials studied is proposed. The manufacturing process followed for the production of the panels is shown schematically in Figure 12.

In this way, an environmentally friendly product design is presented for use in the construction sector. The aim is to reduce the consumption of natural resources and chemical agents used in the production of prefabricated products and to show new ways of revaluing ELTs. Its simple manufacturing process makes it possible to move towards an industrialisation of the construction sector, which is committed to the development of modular parts with a better design adjustment that generates less waste on site [51].

**Figure 13:**
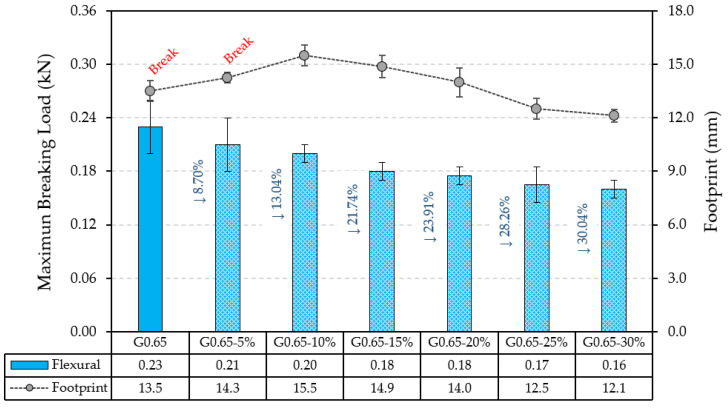
Plate bending test and impact toughness test results.

Figure 13 shows the tests performed for plate bending strength and impact toughness.

Firstly, as can be seen in Figure 13, the maximum ultimate load of the plates decreased as the content of recycled material increased. These results are in agreement with those obtained in the bending strength test according to UNE-EN 13279-2 shown in Figure 6. Thus, for the sample with the highest raw material substitution by ELT aggregates (G0.65-30%), there was a decrease of 30.04% in the bending strength.

This effect on the decrease in the flexural strength of plaster boards by incorporating plastic waste in the matrix of the composites has been observed previously by other researchers. Thus, Pedreño-Rojas et al. observed a decrease in the flexural strength of gypsum plates with the incorporation of polycarbonate waste from CD and DVD wastes [52]. Similarly, Vidales-Barriguete observed a similar effect in his doctoral thesis by replacing gypsum material in volume with plastic re-waste from low-voltage cables [49].

However, Figure 13 shows the positive effect of these rubber residues in cushioning the impact on the surface of the plaster composites. From a partial substitution of 10% by volume with ELT aggregates, the footprint diameter is progressively reduced. Furthermore, it could be observed that the reference and G0.65-5% plates, although with a reduced footprint diameter, fractured during the impact test because of their less elastic behaviour. For this reason, it is understood that the incorporation of these ELT residues in prefabricated plaster can be favourable in improving the impact toughness, in line with the results obtained in the research carried out by Herrero et al. [46].

**Figure 14:**
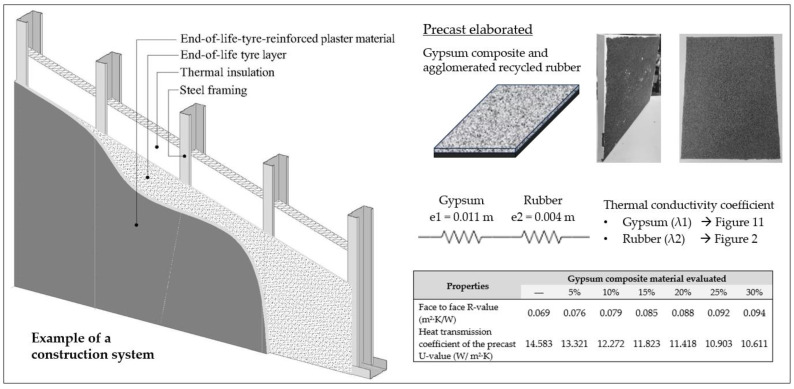
Example of a construction system with the panels developed, and determination of their thermal properties.

Lastly, Figure 14 shows the values collected for the thermal resistance and heat transmission coefficient of the prefabricated elements studied. Additionally, a construction detail of their possible application in partition walls is included.

As can be seen in Figure 14, the total thermal resistance of the prefabricated products increases as the content of recycled rubber aggregates added increases, and a beneficial effect derived from the incorporation of the agglomerated recycled rubber band is observed. These mixed prefabricated products have a design that can be commercialised in the building sector, with a higher environmental quality than other gypsum composites for similar applications. In addition, besides improving energy efficiency by reducing the thermal conductivity of the partition cladding materials, the incorporation of the agglomerated recycled rubber band mitigates the thermal bridges generated between the prefabricated panel and the supporting metal studs. These thermal bridges are a source of heat losses that have been studied in other investigations, in which the beneficial effect of the agglomerated recycled rubber as thermal break strips in the partitions has been observed [53,54]. Moreover, given the good acoustic-related properties of the agglomerated recycled rubber, e.g., high compressibility rate (Figure 2), a higher vibration attenuation leading to an increase of the airborne sound insulation provided by this new partition wall system is expected.

However, these types of panels, by incorporating the thermal bridge break in their manufacturing process, represent an improvement in terms of ease of execution and reduction of assembly times. Thus, a creative solution is proposed to reformulate the design of prefabricated panels for interior partition walls, extending the use of secondary raw materials, being technically possible to develop and having analysed their properties. In this way, a differentiated product is being offered that would allow the companies involved in its development to opt for the achievement of competitive advantages in costs and product differentiation.

## 4. Conclusions

In this work, the physical and mechanical behaviours of gypsum composites with partial substitution of the original raw material by ELT aggregates with sizes between 0 and 0.8 mm were studied. In the same way, a critical discussion of the results was carried out, and the properties of a new prefabricated prototype of our own design were analysed, which aim to meet the needs of customers with a greater environmental awareness and contribute to the current need of construction companies to evolve towards greater sustainability in the sector.

With the specific conclusions of the experimental program developed, the following can be highlighted:

- Gypsum composites were developed with a partial substitution of up to 30% by volume with recycled rubber aggregates. Although it is true that there was a reduction in flexural and compressive strength of up to 29.6% and 41.2%, respectively, in both cases, the minimum strength value required by the current standards was far exceeded, and the surface hardness was higher than 70 Shore C units in all the cases analysed.
- From the SEM study, the correct setting of the gypsum composites produced by the formation of dihydrate crystals was evident, as well as the high degree of cohesion of the matrix and the good adhesion at the interface between the ELT waste and the gypsum base material.
- It was observed that the incorporation of these recycled rubber aggregates reduces water absorption by capillarity, reducing the height reached by water in the composites subjected to this phenomenon by up to 53.6%. These results were consistent with those obtained for open porosity, which were also reduced in the composites with the highest amount of residue.
- The recycled rubber aggregates allowed a slight reduction in the density of the gypsum compounds, originating a significant decrease in the final thermal conductivity of the developed material, which was reduced by up to 39.1% for the G.65-30% compound.
- In order to provide practical application of the research, a prefabricated panel design was proposed that combined the studied gypsum material with bonded rubber bands. With these prefabricated plates, a decrease in the mechanical resistance to bending, an increase in the impact absorption capacity and a decrease in the thermal conductivity of the prefabricated products made with agglomerated rubber bands were deduced. From this, their potential to be applied in the execution of partition walls and light interior partitions was inferred.

In summary, it is considered that this research contributes in a relevant and original way to the recovery and revalorisation of waste from ELT, extending the useful life of these subproducts and improving the circularity of construction materials. Finally, as limitations of this work that allow establishing future lines of research based on the results obtained, it is worth mentioning the absence of acoustic and fire performance tests on the materials developed. These complementary tests would be necessary for the possible commercialisation of the products designed in this work and could be complemented with a life cycle analysis of the final prefabricated product.

## Figures and Tables

**Table 1:** Studies carried out with gypsum composites and the incorporation of rubber aggregates in the last three years (2021–2023).

Ref.	Rubber Type Added	Addition Percentage	Properties Analysed ^(^*^)^									
			A	B	C	D	E	F	G	H	I	J
[20]	ELT aggregates 0.063–8.0 mm	5% by weight and carbon fibres	•	•	•				•	•		
[21]	ELT aggregates 0.0–5.0 mm	14.5–46.6% by volume	•	•	•				•		•	•
[22]	Natural rubber latex	10–20–30–40% by weight	•	•		•	•		•			•
[23]	Natural rubber latex	20–50% by weight	•	•		•						•
[24]	ELT aggregates 4.0–2.5–0.5 mm	15% by weight and polymers	•		•	•	•		•		•	
[25]	ELT aggregates 2.5–4.0 mm	8.8–17.6% by weight and fibres	•	•	•	•		•	•			
[26]	ELT aggregates 2.96–3.60 mm	5–10–20% by weight	•				•		•			
[27]	ELT aggregates 2.5–4.0 mm	10–20–30% by weight	•		•	•		•	•			

^(^*^)^ A: flexural strength; B: compressive strength; C: superficial hardness; D: thermal behaviour; E: acoustic properties; F: water properties; G: density; H: longitudinal elasticity modulus; I: fire resistance; J: chemical composition.

**Table 2:** Properties of type B1 building gypsum (provided by the manufacturer [31]).

Purity (%)	Particle Size (mm)	Flexural Strength (MPa)	Compressive Strength (MPa)	pH	Fire-Resistant UNE-EN 13501-1:2019 [32]	Water Vapour Diffusion (μ)
>80	0–0.2	≥1	≥2	>6	A1	6

**Table 3:** Proportions used for the plaster compound dosage.

Sample	Proportions by Weight (g)			Proportions by Volume (%)		
	Plaster	Water	Rubber	Plaster	Water	Rubber
G0.65	1000.0	650.0	―	60.6	39.4	―
G0.65-5%	950.0	617.5	25.0	57.6	37.4	5.0
G0.65-10%	900.0	585.0	50.0	54.6	35.4	10.0
G0.65-15%	850.0	552.5	75.0	51.6	33.4	15.0
G0.65-20%	800.0	520.0	100.0	48.6	31.4	20.0
G0.65-25%	750.0	487.5	125.0	45.6	29.4	25.0
G0.65-30%	700.0	455.0	150.0	42.6	27.4	30.0

**Table 4:** Results for the total water absorption and open porosity of the composites produced.

Type	G0.65	G.065-5%	G0.65-10%	G0.65-15%	G0.65-20%	G0.65-25%	G0.65-30%
Total Water absorption (%)	43.36	42.54	40.03	39.04	38.18	37.01	36.67
Open Porosity (%)	45.22	44.67	42.17	39.53	39.81	38.60	38.19

## Data Availability

Data are contained within the article.
